# Pixel-level annotated dataset of computed tomography angiography images of acute pulmonary embolism

**DOI:** 10.1038/s41597-023-02374-x

**Published:** 2023-08-04

**Authors:** João Mario Clementin de Andrade, Gabriel Olescki, Dante Luiz Escuissato, Lucas Ferrari Oliveira, Ana Carolina Nicolleti Basso, Gabriel Lucca Salvador

**Affiliations:** 1 Department of Radiology and Image Diagnosis, Hospital de Clínicas, Federal University of Paraná, Curitiba, Brazil; 2 Department of Informatics, Federal University of Paraná, Curitiba, Brazil

**Keywords:** Databases, Diagnosis, Scientific data, Computer science, Medical research

## Abstract

Pulmonary embolism has a high incidence and mortality, especially if undiagnosed. The examination of choice for diagnosing the disease is computed tomography pulmonary angiography. As many factors can lead to misinterpretations and diagnostic errors, different groups are utilizing deep learning methods to help improve this process. The diagnostic accuracy of these methods tends to increase by augmenting the training dataset. Deep learning methods can potentially benefit from the use of images acquired with devices from different vendors. To the best of our knowledge, we have developed the first public dataset annotated at the pixel and image levels and the first pixel-level annotated dataset to contain examinations performed with equipment from Toshiba and GE. This dataset includes 40 examinations, half performed with each piece of equipment, representing samples from two medical services. We also included measurements related to the cardiac and circulatory consequences of pulmonary embolism. We encourage the use of this dataset to develop, evaluate and compare the performance of new AI algorithms designed to diagnose PE.

## Background & Summary

Pulmonary embolism (PE) has a high incidence and mortality. It occurs when a blood clot, most commonly from the deep venous system, moves into the pulmonary arterial circulation^1^. Up to 300,000 deaths per year are estimated to occur in the United States due to PE^2^. Less than 10% of deaths occur among diagnosed and treated patients, indicating a potential reduction in mortality by improving the diagnostic accuracy for the disease^3^.

Computed tomography pulmonary angiography (CTPA) is the examination of choice for evaluating patients with PE^4,5^. After intravenous infusion of iodinated contrast medium, CT is performed when there is optimal opacification of the pulmonary arterial circulation, and the thrombus is identified as an intraluminal filling defect.

CTPA image interpretation is a complex task: radiologists must carefully search for contrast filling defects in the entire pulmonary arterial vasculature across a large number of images. Technical problems, patient-related factors, anatomical issues and the presence of other pathologies^6^ can lead to misdiagnosis.

Computer-aided diagnosis (CAD) programs aimed at reducing these errors can reduce mortality. Several approaches have already been proposed^7–48^; however, a definitive solution has not yet been reached. More recently, there has been increased interest in the creation of artificial intelligence (AI) techniques, especially using artificial neural networks (ANNs), for addressing this problem.

The diagnostic performance of these techniques is highly dependent on the dataset used for training them, as it must contain examinations as diverse as those in real applications. The diagnostic accuracy of these methods tends to increase by augmenting the training dataset^49^.

Obtaining reliable datasets is a considerable obstacle encountered by researchers, as it is time-consuming, requires radiologists with experience to recognize PE and depends on medical center cooperation. Furthermore, to be suitable for supervised learning applications, the dataset must be annotated. Two different annotation approaches have been used in the three public datasets available: a pixel-level annotation, in which all pixels of the thrombus are demarcated as a ground truth, and annotations at the image and study levels^50^, in which images with a visible PE receive a label, but the thrombus itself is not demarcated. The first approach is more versatile and can be converted into an annotation at the image level, but the opposite is not true.

The pixel-level annotation may help the algorithm to predict the exact region in which the embolus is located and to verify if this was the region used by the algorithm to generate the diagnostic output. It is especially useful for evaluating CADs designed to diagnose PE, as a high false-positive rate is a notable challenge for many of these algorithms.

To date, there are only two public datasets containing CTPA examinations with pulmonary emboli annotated at the pixel level. The first one contains 91 examinations of patients with PE obtained using SIEMENS CT scanners^51^, and the second contains 35 examinations, 33 of which were conducted in patients with PE, obtained with CT scanners from PHILIPS and Neusoft Medical System Co^52^. The information published from these datasets does not disclose the examination selection process or the inclusion and exclusion criteria.

A recently published guide for research on AI^53^ highlight*s* the importance of using datasets containing images from devices from multiple vendors due to the variability inherent in such images.

There is a shortage of public datasets with a representative sample of examinations annotated at the pixel level that the AI algorithm can process in a real clinical setting. In medical practice, there is great variability among CTPA images, which occurs due to patient-related factors, such as different biotypes and comorbidities that can obscure PE, and technical factors, such as delays in image acquisition following infusion of the contrast media or with inadequate infusion flow, that lead to suboptimal image quality.

**Fig. 1:**
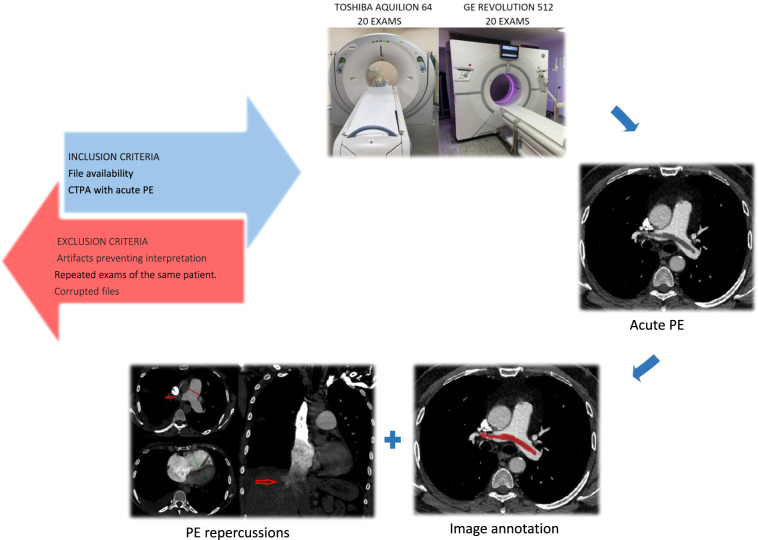
Steps for producing the dataset. First step: definition of inclusion and exclusion criteria. Second and third steps: selection of 20 examinations performed with each machine on patients with acute PE. Fourth step: image annotation at the pixel and image levels. Fifth step: evaluation of features related to right heart strain and pulmonary artery hypertension.

We developed a dataset with a sample of cases of acute PE annotated at the pixel and image levels, making it suitable for algorithms developed using both approaches (Fig. 1). Our dataset contains 40 examinations performed with multidetector scanners, half from a Toshiba CT and the other half from a GE CT^54^.

The dataset was primarily used in conjunction with the two public datasets of CTPA examinations with pulmonary emboli annotated at the pixel level^51,52^ to develop a program for the diagnosis of PE^48^. A method capable of finding and segmenting PEs in CT images using deep learning was subsequently developed.

We encourage the use of this dataset to develop, evaluate and compare the performance of AI algorithms designed to diagnose PE.

## Methods

The study was approved by the Research Ethics Committee of Hospital de Clínicas-Federal University of Paraná. Given the retrospective nature of the study, only retrospective access to anonymized scan files was necessary, and so the need for informed consent was waived by the ethics committee. Aiming to preserve the identity of the participants, the examinations were deidentified by deleting patients’ personal information, such as names, date of birth and identification numbers from the CT scans. Fields corresponding to time or numbers were replaced by “000000.00”. Fields corresponding to dates were replaced by “00010101”. Written Fields were replaced by “Anonymized” or removed.

### Imaging

Twenty CTPA scans were performed in a public university hospital with a 64-channel Toshiba Aquilion CT scanner, with a tube voltage of 120 KVp, slice thickness of 1.0 mm, gantry rotation time of 0.5 seconds, beam pitch of 1.485 and dose modulation protocol.

The other twenty scans were performed in a private imaging practice with a GE Revolution 512 CT scanner, with a tube voltage of 120 KVp, slice thickness of 0.625 mm, slice interval slice thickness of 0.625 mm, gantry rotation time of 0.5 seconds, beam pitch of 0.9 and dose modulation protocol.

### Image segmentation

All CTPA scans were used to make a diagnosis of acute PE by a staff radiologist. The diagnosis and location of the acute pulmonary embolism were confirmed by a thoracic radiologist with 32 years of experience. After this, a third-year resident generated the pixel-level ground-truth masks, which were revised by a certified radiologist.

**Fig. 2:**
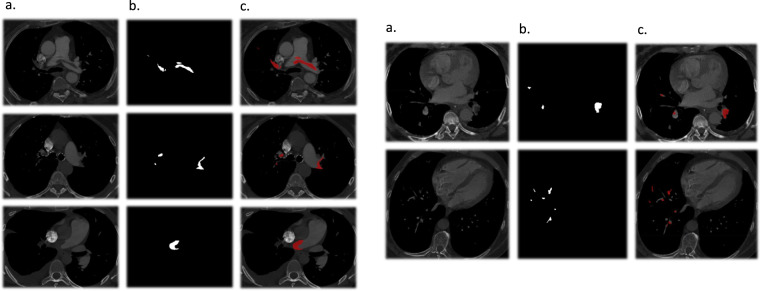
Pixel level annotation. Column (**a**) shows CTPA images of pulmonary emboli in different anatomical locations. In column (**b**), pixel-level annotations show all pixels of the embolus of the corresponding CTPA image in column (**a**) in white. In column (**c**), the corresponding images of columns (**a**) and (**b**) are superimposed, and the thrombus is shown in red.

The examinations were segmented using manual mode in ITK-SNAP^55^, generating the ground-truth mask in which the thrombus pixels are demarcated (Fig. 2). Based on this mask, image-level segmentation was performed, labeling slices containing the thrombi.

### CTPA features related to right heart strain and pulmonary artery hypertension

**Fig. 3:**
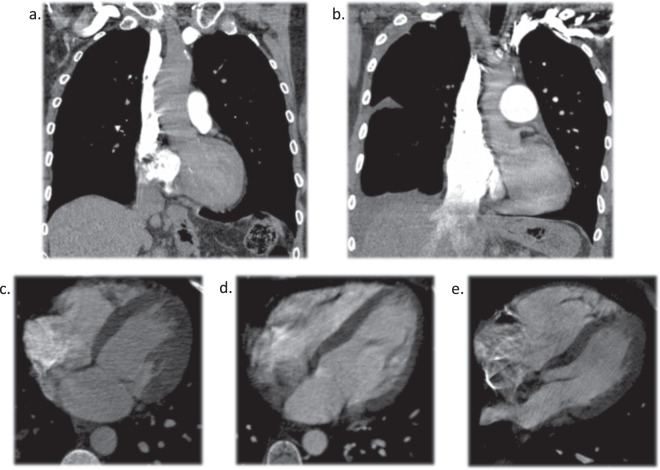
CTPA features related to right heart strain and pulmonary artery hypertension. (**a**) Exam TS04 without IVC reflux. (**b**) Exam TS19 with IVC reflux. (**c**) Exam TS19 showing the interventricular septum in its normal position. (**d**) Exam TS02 showing a flattened interventricular septum. (**e**) Exam TS10 showing paradoxical interventricular septal bowing.

Obstruction of the pulmonary vasculature due to PE can increase vascular resistance, leading to an increase in pulmonary arterial pressure and right cardiac strain. Indirect signs such as pulmonary artery dilatation, right ventricular enlargement (increase in the right ventricle-to-left ventricle diameter ratio), inferior vena cava (IVC) contrast reflux, and abnormal positioning of the interventricular septum (flattening or even paradoxically bowing toward the left ventricle), can be observed on CTPA scans (Fig. 3).

In all CTPA scans, we evaluated the largest artery involved, inferior vena cava reflux, interventricular septum flattening or paradoxical bowing, pulmonary artery trunk diameter (PAD), transverse diameters of the right ventricle (RV) and left ventricle (LV)—measured between the endocardial surfaces in the largest place perpendicular to the longitudinal axis—and right ventricle-to-left ventricle diameter ratio (Tables 1, 2).

**Table 1:** Data from exams obtained with the Toshiba Aquilion 64.

Case	Sex	Age	PAD (mm)	RV (mm)	LV (mm)	RV/LV	IVC Reflux	IV Septum	Largest affected vessel
1	F	63	24	43	57	0.75	Present	Normal	Lobar
2	F	11	20	37	40	0.92	Absent	Flattened	Trunk bifurcation
3	F	77	29	45	35	1.29	Absent	Flattened	Main pulmonary artery
4	F	38	34	52	29	1.79	Present	Paradoxically bowed	Main pulmonary artery
5	F	68	29	41	43	0.95	Absent	Flattened	Trunk bifurcation
6	F	54	28	39	54	0.72	Present	Flattened	Main pulmonary artery
7	F	58	35	33	35	0.94	Present	Normal	Lobar
8	F	64	31	37	48	0.77	Absent	Flattened	Segmental
9	M	29	28	23	31	0.74	Absent	Flattened	Segmental
10	M	68	26	52	35	1.49	Present	Paradoxically bowed	Main pulmonary artery
11	F	81	27	29	42	0.69	Present	Normal	Lobar
12	F	84	31	52	41	1.27	Present	Normal	Trunk bifurcation
13	F	41	24	32	38	0.84	Absent	Normal	Segmental
14	F	48	34	43	51	0.84	Present	Normal	Segmental
15	F	45	20	40	45	0.89	Absent	Normal	Main pulmonary artery
16	F	75	31	33	46	0.72	Present	Normal	Subsegmental
17	F	59	31	42	36	1.17	Present	Flattened	Trunk bifurcation
18	F	42	24	42	45	0.93	Absent	Normal	Segmental
19	F	26	25	35	50	0.70	Absent	Normal	Lobar
20	F	50	25	34	52	0.66	Absent	Normal	Main pulmonary artery

F: female. M: male. PAD: pulmonary artery diameter. RV: right ventricle diameter. LV: left ventricle diameter. RV/LV: right ventricle-to-left ventricle diameter ratio. IVC Reflux: inferior vena cava reflux of contrast media. IV Septum: interventricular septum position.

**Table 2:** Data from exams obtained with the GE Revolution 512.

Case	Sex	Age	PAD (mm)	RV (mm)	LV (mm)	RV/LV	IVC Reflux	IV Septum	Largest affected vessel
1	M	59	27	52	57	0.91	Absent	Normal	Main Artery (unilateral)
2	F	72	32	60	40	1.50	Absent	Flattened	Main Artery (unilateral)
3	M	71	36	38	32	1.19	Present	Normal	Lobar
4	M	62	28	33	34	0.97	Absent	Flattened	Trunk bifurcation
5	M	73	30	32	42	0.76	Absent	Normal	Segmental
6	M	82	40	47	30	1.57	Present	Flattened	Trunk bifurcation
7	F	21	32	39	47	0.83	Absent	Normal	Segmental
8	F	82	29	32	42	0.76	Absent	Normal	Segmental
9	M	50	28	22	40	0.55	Present	Normal	Lobar
10	F	57	26	55	38	1.45	Absent	Paradoxically bowed	Subsegmental
11	M	31	27	25	51	0.49	Absent	Normal	Segmental
12	F	36	22	30	35	0.86	Absent	Normal	Lobar
13	F	86	26	35	36	0.97	Present	Normal	Subsegmental
14	F	57	38	50	48	1.04	Present	Flattened	Main Artery (unilateral)
15	F	32	27	44	42	1.05	Absent	Flattened	Segmental
16	M	38	30	46	50	0.92	Absent	Normal	Lobar
17	F	81	31	50	50	1.00	Present	Normal	Lobar
18	M	54	28	44	50	0.88	Absent	Normal	Lobar
19	M	41	29	40	49	0.82	Absent	Normal	Trunk bifurcation
20	M	54	29	38	64	0.59	Present	Normal	Segmental

F: female. M: male. PAD: pulmonary artery diameter. RV: right ventricle diameter. LV: left ventricle diameter. RV/LV: right ventricle-to-left ventricle diameter ratio. IVC Reflux: inferior vena cava reflux of contrast media. IV Septum: interventricular septum position.

## Data Records

All data records described in this paper are available on a Figshare repository^54^. This repository contains three folders. The first contains CTPA images in Digital Imaging and Communications in Medicine (DICOM) format. The second contains the ground-truth pixel-level segmentation of the location of the pulmonary embolus in Neuroimaging Informatics Technology Initiative (NiFTI) format. The segmentations consist of a three-dimensional matrix in which each element corresponds to a voxel of the CT scan: the elements corresponding to the embolus have a value of “1”, and the others have a value of “0”. The third is in comma separate values (CSV) format, in which each element corresponds to a slice of the CT scan. The first element represents the first slice, and the last element represents the lowest slice. Slices in which the embolus can be visualized are represented by the number “1”, and the others by the number “0”.

The files corresponding to patients scanned with the GE equipment are named 01GE to 20GE, and the files corresponding to patients scanned with the Toshiba equipment are named 01TS to 20TS, according to the number of each patient in Tables 1, 2, which are also available on the repository in Excel binary file (XLS) format.

## Technical Validation

### Exam selection

The CTPA scans were selected retrospectively by a search of the digital files from a public university hospital and a private imaging practice.

An arbitrary starting date was defined for each medical facility. From the defined starting date, all CTPA scans were sequentially reviewed until we reached 20 examinations with PE from each device that fit the inclusion and exclusion criteria.

For the Toshiba device, the examinations were performed from November 5, 2018 to February 5, 2019. For the GE device, the examinations were performed from November 9, 2018 to September 20, 2019.

### Inclusion criteria

Chest CT scans performed using the PE protocol (CTPA) for diagnosing acute PE. File availability in the Picture Archive and Communication System (PACS) from each medical center.

### Exclusion criteria

Artifacts that prevented the radiologist from visually interpreting the images. Examinations with fully or partially corrupted files. Follow-up examinations (only the first CTPA was used).

There was no restriction on age, patient status (inpatients or outpatients) or any other inclusion or exclusion criteria different from those mentioned.

## Data Availability

All code for loading and normalization of the dataset is available in GitHub (https://github.com/glescki/dicom_image_parser). For parsing the data, the PyDicom library is recommended, and the loading of the labels can be performed with a parser available in GitHub. Each DICOM file should be loaded separately and then joined within a data structure. For normalization, it is recommended that the spacing in the z-axis of all slices be changed to 1.
